# Tumor-derived granulocyte colony-stimulating factor diminishes efficacy of breast tumor cell vaccines

**DOI:** 10.1186/s13058-018-1054-3

**Published:** 2018-10-22

**Authors:** Sruthi Ravindranathan, Khue G. Nguyen, Samantha L. Kurtz, Haven N. Frazier, Sean G. Smith, Bhanu prasanth Koppolu, Narasimhan Rajaram, David A. Zaharoff

**Affiliations:** 1 Department of Biomedical Engineering, University of Arkansas, Fayetteville, AR USA; 2 Cell and Molecular Biology Program, University of Arkansas, Fayetteville, AR USA; 3 Department of Microbiology and Immunology, University of North Carolina, Chapel Hill, NC USA; 4 Honors College, University of Arkansas, Fayetteville, AR USA; 5 Joint Department of Biomedical Engineering, University of North Carolina, Chapel Hill, NC and North Carolina State University, Raleigh, NC USA

**Keywords:** Breast cancer vaccine, Autologous tumor cell vaccine, MDSCs, Breast cancer immunogenicity

## Abstract

**Background:**

Although metastasis is ultimately responsible for about 90% of breast cancer mortality, the vast majority of breast-cancer-related deaths are due to progressive recurrences from non-metastatic disease. Current adjuvant therapies are unable to prevent progressive recurrences for a significant fraction of patients with breast cancer. Autologous tumor cell vaccines (ATCVs) are a safe and potentially useful strategy to prevent breast cancer recurrence, in a personalized and patient-specific manner, following standard-of-care tumor resection. Given the high intra-patient and inter-patient heterogeneity in breast cancer, it is important to understand which factors influence the immunogenicity of breast tumor cells in order to maximize ATCV effectiveness.

**Methods:**

The relative immunogenicity of two murine breast carcinomas, 4T1 and EMT6, were compared in a prophylactic vaccination-tumor challenge model. Differences in cell surface expression of antigen-presentation-related and costimulatory molecules were compared along with immunosuppressive cytokine production. CRISPR/Cas9 technology was used to modulate tumor-derived cytokine secretion. The impacts of cytokine deletion on splenomegaly, myeloid-derived suppressor cell (MDSC) accumulation and ATCV immunogenicity were assessed.

**Results:**

Mice vaccinated with an EMT6 vaccine exhibited significantly greater protective immunity than mice vaccinated with a 4T1 vaccine. Hybrid vaccination studies revealed that the 4T1 vaccination induced both local and systemic immune impairments. Although there were significant differences between EMT6 and 4T1 in the expression of costimulatory molecules, major disparities in the secretion of immunosuppressive cytokines likely accounts for differences in immunogenicity between the cell lines. Ablation of one cytokine in particular, granulocyte-colony stimulating factor (G-CSF), reversed MDSC accumulation and splenomegaly in the 4T1 model. Furthermore, G-CSF inhibition enhanced the immunogenicity of a 4T1-based vaccine to the extent that all vaccinated mice developed complete protective immunity.

**Conclusions:**

Breast cancer cells that express high levels of G-CSF have the potential to diminish or abrogate the efficacy of breast cancer ATCVs. Fortunately, this study demonstrates that genetic ablation of immunosuppressive cytokines, such as G-CSF, can enhance the immunogenicity of breast cancer cell-based vaccines. Strategies that combine inhibition of immunosuppressive factors with immune stimulatory co-formulations already under development may help ATCVs reach their full potential.

**Electronic supplementary material:**

The online version of this article (10.1186/s13058-018-1054-3) contains supplementary material, which is available to authorized users.

## Background

In 2018, approximately 41,400 breast-cancer-related deaths will occur in the USA [1]. About 90% of these deaths will be due to metastases. Since only about 4% of the 265,000+ new patients with breast cancer are typically diagnosed with stage IV metastatic cancer, the vast majority of breast-cancer-related deaths are due to the recurrence and progression of breast cancers initially diagnosed at stages I–III. In an attempt to prevent tumor recurrence, approximately four out of every five patients with breast cancer receive adjuvant therapies such as chemotherapy, hormone therapy, and/or radiotherapy following tumor resection [2]. Even with state-of-the-art adjuvant treatments, the 5-year recurrence rates for stage I, II, and III breast cancer are 7%, 11%, and 13%, respectively [3]. After 10 years, the overall breast cancer recurrence rate increases to 20% [3]. Furthermore, side effects associated with current adjuvant therapies can be life-altering and even life-threatening [4]. Thus, strategies capable of more effectively and more safely preventing progressive breast cancer recurrences, particularly after standard-of-care tumor resection, are urgently needed.

Adjuvant breast cancer vaccines are of interest due to their potential to educate a patient’s immune system to recognize and eliminate occult tumor cells before a recurrence can develop. In particular, autologous tumor cell vaccines (ATCVs) comprise a promising class of vaccines capable of inducing personalized, polyclonal anti-tumor immune responses [5–14]. Patient/tumor-specific polyclonal immune responses are especially relevant for breast cancer with high intra-patient and inter-patient molecular heterogeneity that facilitates resistance to targeted therapies [15–19]. Because ATCVs are generated from a patient’s own malignant cells, they present a complete and personalized library of tumor-associated antigens (TAAs). In contrast, peptide-based vaccines deliver one or a couple different peptides and are prone to tumor escape through downregulation of the targeted epitope(s). Furthermore, since ATCVs are “antigen agnostic,” they could be used in the management of any subtype of breast cancer including triple-negative breast cancers (TNBCs), which lack hormone and human epidermal growth factor receptor 2 (HER2) receptors, the usual targets for breast cancer therapies.

While ATCVs have been shown to be safe and active in numerous clinical studies, a major barrier to their widespread clinical use is inconsistent, if not limited, immunogenicity. Patient-derived cancer cells, which form the basis of the vaccine, have undergone extensive immunoediting to avoid elimination by the host’s immune system [20]. Common mechanisms that cancer cells use during immune escape include (1) downregulation of major histocompatibility complex (MHC I/II) molecules and development of defects in antigen presentation; (2) downregulation of costimulatory molecules, such as B7–1 and B7–2; (3) upregulation of immunoinhibitory molecules, such as programmed death-ligand 1 (PD-L1); (4) loss or modification of tumor-associated antigen(s); and (5) increased production of immunosuppressive factors such as indoleamine 2,3-dioxygenase (IDO), IL-10 and tumor growth factor (TGF)β [21]. As a result, nearly all ATCVs currently under development utilize strategies to boost tumor cell immunogenicity through one or more of the following: transfection of autologous tumor cells with costimulatory molecules [22–26], conjugation of immunostimulatory moieties to autologous tumor cells [10, 27]; co-formulation with immunostimulatory molecules [6, 8, 27–30]; or engineering autologous tumor cells to secrete adjuvant cytokines [9, 31–41]. Employing these strategies has demonstrated significant increases in antitumor immunity against various malignancies in clinical studies [8–10, 26, 27, 33, 36, 37, 39, 41–43].

For breast cancer, ATCV clinical studies have been limited to three completed [44–46] and two active trials [47, 48]. All three completed studies show promise in generating antitumor responses [49]. Despite the relatively small number of clinical studies, breast cancer remains an ideal indication for ATCV deployment as (1) 62% of breast cancer cases are diagnosed at stage I, where the tumor is still localized in the breast with minimal impact on the patient’s immune status [50]; (2) nearly all patients with breast cancer undergo tumor resection, thus ensuring a source of tumor cells for ATCV production; and (3) the vast majority of patients with breast cancer have minimal, if any, detectable disease after resection so the tumor burden is low.

Because of the aforementioned heterogeneity in breast cancer, it is expected that breast ATCVs will display varying degrees of immunogenicity. Thus, the goal of this study was to begin to define the primary determinants of ATCV immunogenicity by comparing two murine models of breast adenocarcinoma, 4T1 and EMT6: 4T1 is a poorly immunogenic murine breast cancer cell line that shares many features with human stage IV breast cancer [51–53]. EMT6 on the other hand, is a highly aggressive, yet immunogenic cell line [54–56]. By understanding the key drivers of breast cancer immunogenicity, we may be able to directly and more efficiently enhance ATCVs during ex vivo modifications. At the very least, data gathered could be used to identify which patients are better candidates for adjuvant ATCV therapy. During the study, we observed that myeloid-derived suppressor cells (MDSCs) played a dominant role in influencing breast ATCV immunogenicity. The immunosuppressive role of MDSCs in breast cancer progression and metastasis is well-documented [57–60]. In particular, the levels of circulating MDSCs were found to correlate with clinical stage and metastatic tumor burden [61]. However, to the best of our knowledge, the influence of MDSCs on ATCV efficacy has not been explored. Thus, the focus of the latter stages of this study shifted towards identifying and blocking the origin of breast-cancer-related MDSCs as a strategy to enhance ATCV immunogenicity.

## Methods

### Cell culture

Murine breast adenocarcinoma cells 4T1 and EMT6 were purchased from American Type Culture Collection (Manassas, VA, USA). The rest of the breast cancer cells, namely 4T07, 67NR, 66Cl4, 168FARN were a generous gift from Dr Fred Miller (Karmanos Cancer Institute, Detroit, MI, USA). All cell lines except EMT6 cells were maintained in Dulbecco’s modified eagle medium (DMEM), supplemented with 10% fetal bovine serum (FBS) and 1% penicillin/streptomycin (P/S). EMT6 cells were maintained in Roswell Park Memorial Institute-1640 (RPMI-1640) medium, supplemented with 15% FBS and 1% P/S. All cells were cultured at 37 °C in a humidified incubator with 5% CO_2_.

### Mice

All experimental procedures were approved by the Institutional Animal Care and Use Committee at University of Arkansas. Female Balb/cByJ mice were purchased from The Jackson Laboratory (Bar Harbor, ME, USA) and were housed in microisolator cages. Mice were utilized for experiments at 8–12 weeks of age and animal care followed *The Guide for Care and Use of Laboratory Animals* (National Research Council).

### In vitro proliferation assay

The 4T1 and EMT6 cells were irradiated at 0, 20, 40, 60, 80, or 100 Gy using a Gammacell 1000 cesium irradiator. Cells were then plated in triplicate on a 96-well plate and incubated at 37 °C for 24, 48, 72, or 96 h. After incubation, 20 μl of CellTiter 96 Aqueous One Solution Reagent from Promega (Madison, WI, USA) was added to each well and incubated for another hour. Using a Biotek Synergy 2 plate reader from Biotek Instruments Inc. (Winooski, VT, USA), absorbance was measured at 490 nm and compared to the absorbance of similarly treated known numbers of irradiated 4T1/EMT6 cells to determine the number of viable cells in the sample wells.

### Expression of MHC and costimulatory molecules

Irradiated (100 Gy) and non-irradiated 4T1 and EMT6 cells (5 × 10^5^) were stained with fluorochrome-conjugated anti-CD80 (clone 16-10A1), anti-CD86 (clone GL1), anti-H-2K^b^ (MHC I) (clone AF6–88.5), anti-I-A^d^/I-E^d^ (MHC II) (clone M5/114.15.2), anti-CD54 (ICAM-1) (clone 3E2), and anti-CD95 (FasR) (clone Jo2) (BD Biosciences). Cells were analyzed on a FACSCantoII and differences in median fluorescence intensities (ΔMFI) between unstained and stained cells were determined using FlowJo software (Tree Star, San Carlos, CA, USA).

### In vitro cytokine analysis

The cells (5 × 10^5^ 4T1 or EMT6 cells, untouched or irradiated, and 5 × 10^5^ untouched 4T07, 67NR, 168FARN or 66Cl4 cells) were seeded in separate T25 flasks and cultured for 48 h. Cell culture supernatants were collected and centrifuged to remove any non-adherent cells and stored at − 80 °C until analysis. From the untouched and irradiated 4T1 or EMT6 cells, levels of monocyte-colony stimulating factor (M-CSF), vascular endothelial growth factor (VEGF), transforming growth factor-β (TGF-β), interleukin-6 (IL-6), monocyte chemotactic protein (MCP-1), GM-CSF and G-CSF in cell culture supernatants were quantified. On the other hand, the cell culture supernatants from untouched 4T07, 67NR, 168FARN and 66Cl4 were only evaluated for G-CSF. Levels of M-CSF, VEGF and TGF-β were analyzed using ELISA kits from R&D systems Inc. (Minneapolis, MN, USA) and Biolegend (San Diego, CA, USA). Levels of IL-6, MCP-1, GM-CSF, and G-CSF were analyzed using a cytometric bead array (CBA) on a FACSCantoII from BD Biosciences.

### CRISPR/Cas9 genomic deletion of G-CSF

Using the CRISPR design tool provided by the Zhang laboraoty at Massachussetts Institute of Technology (MIT) (http://crispr.mit.edu/), a 20-bp guide sequence targeting the *G-CSF* gene in 4T1 cells was identified. Guide sequences were cloned into separate pCas-Guide-EF1a-green fluorescent protein (GFP) plasmid via Origene’s cloning service. Plasmids were amplified in *Escherichia coli* and isolated via QIAGEN Plasmid Maxi Kit. For transfection, plasmid encoding guide RNA (gRNA) (10 μg) was mixed with Lipofectamine™ 3000 reagent (ThermoFisher) and added to 1 × 10^6^ 4T1 cells pre-seeded in a 6-well plate. After 48 h, cells expressing GFP were sorted using a FACSAriaIII system (BD Biosciences). Sorted cells were subsequently cloned by limiting dilution. G-CSF expression was quantified by enzyme-linked immunosorbent assay (ELISA) from R&D systems Inc. (Minneapolis, MN, USA). A mixture of clones producing lower than detectable levels of G-CSF were identified and denoted as 4T1.G-CSF^−^.

### Prophylactic vaccination studies

Tumor cell vaccines were generated by irradiating 4T1 or EMT6 cells at 100 Gy using a Gammacell 1000 cesium irradiator. Mice were subcutaneously vaccinated with a primary and booster vaccine 10 days apart, which comprised 1 × 10^6^ irradiated 4T1 cells (4T1 vaccine) or 5 × 10^5^ irradiated EMT6 cells (EMT6 vaccine). For mice in the ipsilateral and contralateral hybrid vaccine groups, 1 × 10^6^ irradiated 4T1 cells and 5 × 10^5^ irradiated EMT6 cells were subcutaneously injected on the same and opposite flanks, respectively. In some instances, where the effect of G-CSF on overall survival was investigated, mice received 4T1.G-CSF^−^ cells in place of 4T1 cells. Further, all vaccinated mice were challenged with 1 × 10^6^ live 4T1, 5 × 10^5^ live EMT6 cells or 1 × 10^6^ live 4T1.G-CSF^−^ cells, 10 days after the booster vaccine. Tumor volumes were recorded 2–3 times per week using the formula:

V = (w × w × l)/2,

where V is tumor volume, w is tumor width and l is tumor length.

### G-CSF in serum from mice

When tumor volumes in mice bearing 4T1, 4T1.GCSF^−^, 4T07, 67NR, 168FARN and 66Cl4 reached about 500 mm^3^, about 400–500 μl of blood was collected in microcentrifuge tubes by submandibular bleeding. After allowing the blood to clot for 30 min at room temperature, samples were centrifuged at 2000 × g for 10 min at 4 °C. The serum was carefully collected from each sample and the levels of G-CSF were determined by ELISA (R&D systems Inc.; Minneapolis, MN, USA).

### Tissue collection and analysis of immune cell subsets

Spleens and draining lymph nodes (DLNs) from 4T1 and 4T1.GCSF^−^ tumor-bearing mice were isolated when tumors reached 500–700 mm^3^. Single cell suspensions were prepared by mechanically dissociating spleen and DLNs with a syringe plunger and passing samples through a 40-μm nylon mesh cell strainer. Splenocytes were additionally treated with ammonium-chloride-potassium buffer (Lonza, Allendale, NJ, USA) for 10 min to lyse red blood cells. Single cell suspensions were then blocked with purified rat anti-mouse CD16/CD32 monoclonal antibody (BD Biosciences) and stained with fluorochrome-conjugated anti-CD11b (clone M1/70), anti-CD19 (clone 1D3), anti-Ly6G and Ly6C (clone RB6-8C5), anti-CD25 (clone PC61), anti-CD4 (clone GK1.5), and anti-CD3ε (clone 145-2C11) (BD Biosciences).

Cells were then rinsed, fixed and permeabilized with 1× Perm/Wash buffer from BD Biosciences. The permeabilized cells were further stained with fluorochrome-conjugated anti-FoxP3 and read on a BD FACSCanto II flow cytometer. Frequencies of MDSCs, T cells, B cells, and regulatory T cells (T_regs_) in the single cell suspensions were determined using FlowJo software (Tree Star, San Carlos, CA, USA). For mice bearing 4T07, 67NR, 66Cl4 and 168FARN tumors, only the spleens were isolated and stained for MDSCs.

### Statistical analysis

All data were analyzed using GraphPad Prism software, version 7 (GraphPad Software, Inc., San Diego, CA, USA). For all in vivo vaccine studies, Kaplan-Meier tumor-free survival curves were plotted and statistical comparisons made using the log rank test. For all other studies, data are represented as mean ± standard deviation. For the experiments that compare cytokine release and expression of MHC and costimulatory molecules by 4T1 and EMT6 before and after irradiation, statistical comparisons were made using two-way analysis of variance (ANOVA) followed by Tukey’s multiple comparison post-hoc test. For experiments where different immune cell subsets in spleen and DLN of mice bearing 4T1 or 4T1.G-CSF^−^ tumors are compared to subsets in naïve mice, statistical comparisons were made using the Kruskal-Wallis test followed by Dunn’s post-hoc test. For all other experiments, statistical comparisons were made using one-way ANOVA followed by Tukey’s post-hoc analysis.

## Results

### Effect of irradiation on proliferation of breast cancer cells

**Fig. 1:**
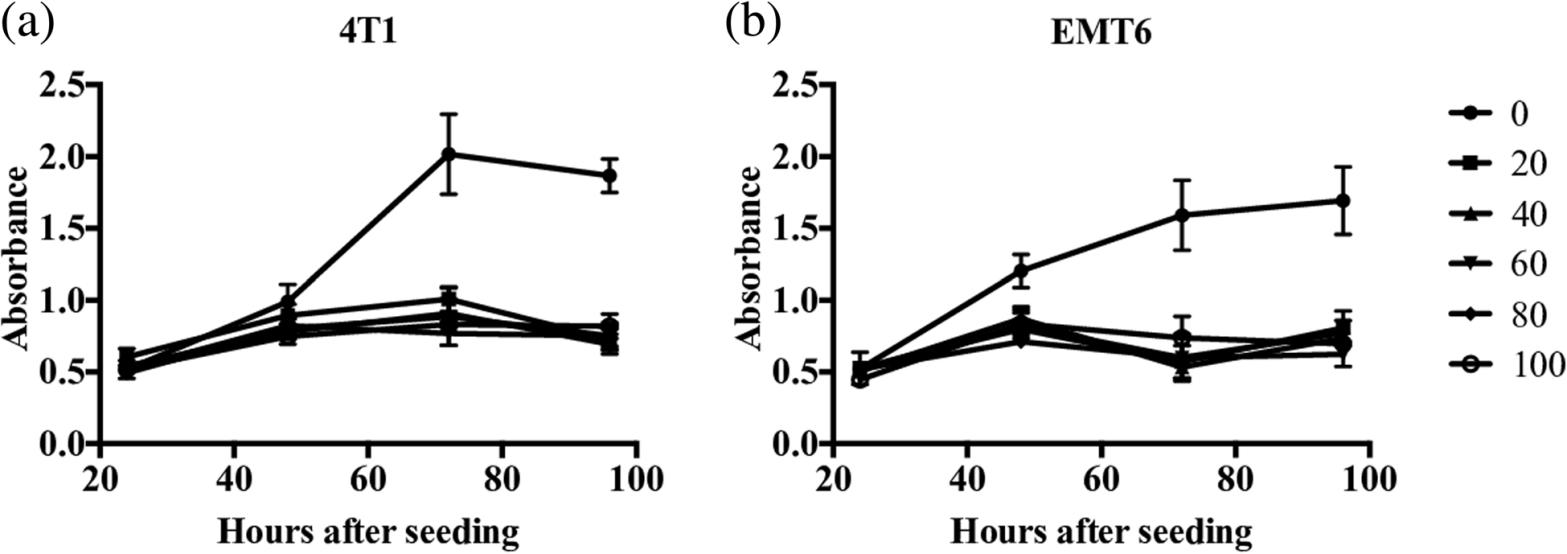
Effect of different doses of irradiation on proliferation of 4T1 and EMT6 cells. The 4T1 (**a**) and EMT6 (**b**) cells were irradiated at 0, 20, 40, 60, 80, or 100 Gy with respective cell culture medium and plated in a 96-well plate with 200 μl of fresh culture medium. After incubating at 37 °C for 24, 48, 72, and 96 h, 20 μl of CellTiter 96 Aqueous One Solution Reagent was added to each well and incubated for an additional 1 h. Number of viable cells in each well was then determined by measuring the absorbance at 490 nm and comparing it with a standard curve generated using known numbers of 4T1 or EMT6 cells. Results are represented as mean ± standard error (**p < 0.01, ***p < 0.001, ****p < 0.0001, one-way analysis of variance with Tukey’s post-hoc analysis)

Prior to using irradiated 4T1 or EMT6 cells as tumor cell vaccines, an appropriate dose of irradiation that effectively prevents tumor cell proliferation was determined using an in-vitro proliferation assay. In the absence of irradiation, both 4T1 and EMT6 cells effectively proliferated over the time observed in this study (24–96 h). However, in the presence of varying doses of irradiation (20–100 Gy), there was no significant difference in viable cell numbers during the study period (Fig. 1).

### Immunogenicity of murine breast carcinoma lines

**Fig. 2:**
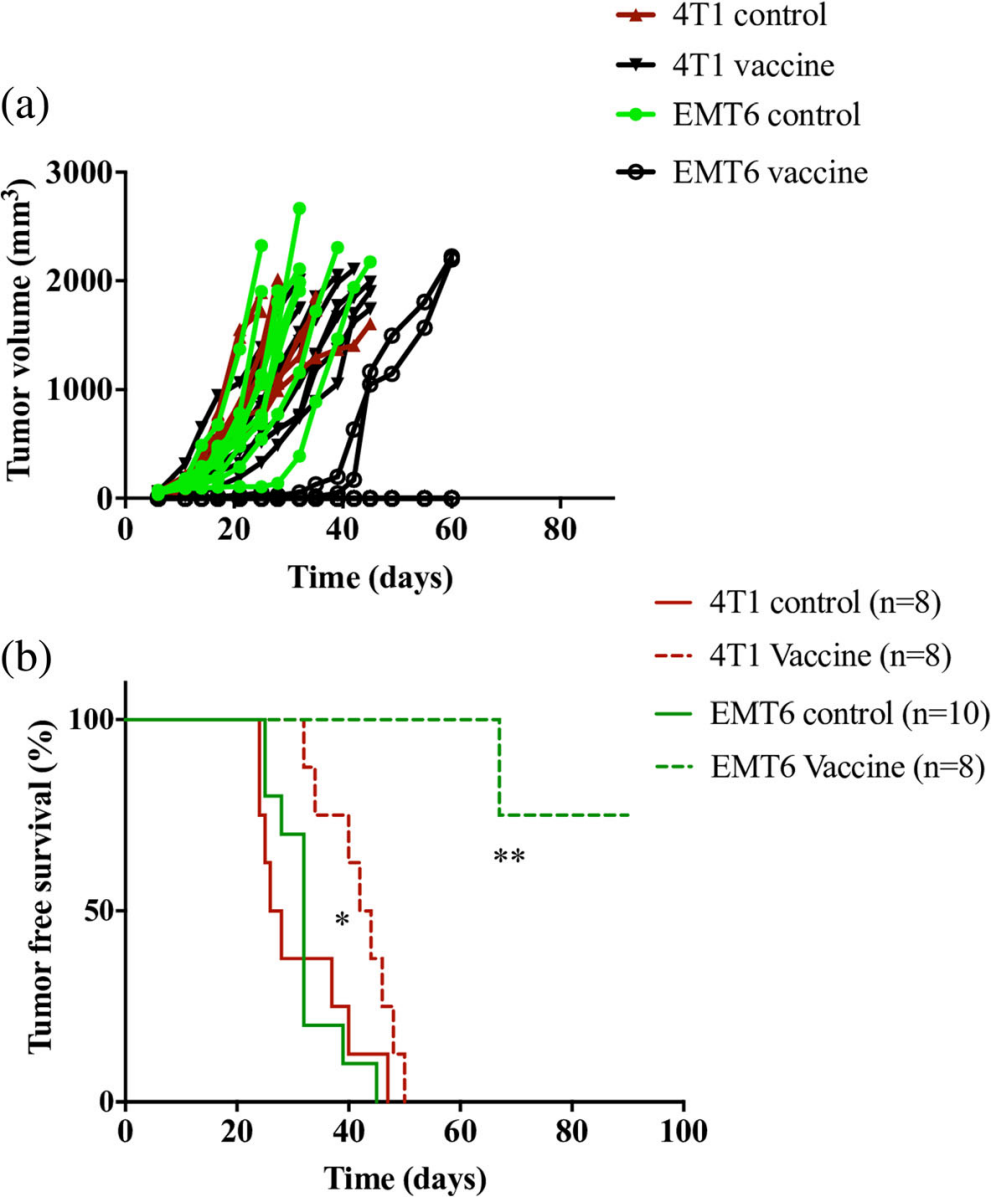
Differences in protective immunity induced by irradiated breast cancer cell lines. Balb/cByJ mice (*n* = 8 per group) received 1 × 10^6^ irradiated 4T1 cells (4T1 vaccine) or 5 × 10^5^ irradiated EMT6 cells (EMT6 vaccine) twice, 10 days apart. Ten days after the booster vaccination, mice were challenged with live 5 × 10^5^ 4T1 or EMT6 cells, respectively. Additionally, naive mice that received only 5 × 10^5^ live EMT6 cells (EMT6 control) or live 4T1 cells (4T1 control) served as controls for each group. Tumor volumes (**a**) and tumor-free survival (**b**) were recorded. (*p < 0.05 vs. naïve/4T1 challenge group, **p < 0.01 vs. naïve/EMT6 challenge group, log rank test)

A standard prophylactic vaccine model was used to evaluate the immunogenicities of 4T1 and EMT6 cells. Mice were vaccinated twice with either irradiated 4T1 or EMT6 cells and challenged with live 4T1 or EMT6 cells, respectively. While all mice in both 4T1 and EMT6 control groups developed tumors, upon vaccination, some of the mice in the EMT6 vaccinated group did not develop any tumor. Moreover, the mice that developed tumors in the EMT6 vaccinated group, showed a delayed tumor incidence when compared to the EMT6 control (Fig. 2a). In both groups, vaccinated mice exhibited some level of protective immunity as demonstrated by extended survival compared to mice in unvaccinated control groups (Fig. 2b). However, EMT6 vaccinated mice exhibited higher overall survival with a *p* value < 0.01 when compared to EMT6 control. On the other hand, 4T1 vaccinated mice had a p value < 0.05 when compared to 4T1 control. Additionally, while all mice in the 4T1 vaccinated group developed and succumbed to tumors within 50 days of tumor inoculation, 75% of the mice in the EMT6 vaccinated group are tumor free survivors.

### Costimulatory molecule and MHC expression on breast cancer cell lines

The elaboration of robust adaptive immunity requires antigen presentation in MHC I or MHC II complexes (signal 1) and simultaneous engagement of costimulatory molecules (signal 2), such as B7–1, B7–2, ICAM-1 and FasR, on APCs, with their cognate receptors, i.e. T cell receptor (TCR), CD28, lymphocyte function-associated antigen 1 (LFA-1) and Fas ligand (Fas-L), on lymphocytes. Thus, MHC and costimulatory molecules on 4T1 and EMT6 cells were evaluated to determine if differences in expression levels could explain the observed differences in immunogenicity. Since irradiated cells displayed higher levels of autofluorescence [62] differences in mean fluorescence intensity values, ΔMFI, between unstained and stained irradiated cells and between unstained and stained non-irradiated cells were analyzed.

Prior to irradiation, 4T1 cells expressed slightly higher levels MHC I when compared to EMT6 cells (4T1: 25.2 ± 3.4; EMT6: 16.6 ± 2.3). However, upon irradiation, EMT6 cells expressed significantly higher level of MHC I which was 49.9 ± 3.3 when compared to 4T1 cells which was only 25.3 ± 3.6. On the other hand, EMT6 cells expressed higher levels of MHC II both before and after irradiation (Table 1).

**Table 1:** Differences in expression of MHC and costimulatory molecules in non-irradiated and irradiated breast cancer cells

	B7–1	B7–2	ICAM-1	MHCI	MHCII	FasR
4T1						
Non-irradiated	6.7 ± 1.3^a^	28.6 ± 6.6	20.6 ± 2.2^a^	25.2 ± 3.4^a^	43.4 ± 1.8	405.3 ± 20.4
Irradiated	14.8 ± 2.8^ab^	50.2 ± 1.4	35.7 ± 4.0^c^	25.3 ± 3.6^a^	133.3 ± 4.2	1073.0 ± 71.6
% change	150 ± 47.1	57.1 ± 19.7	79.3 ± 3.5	11.7 ± 15.4	209.6 ± 31.4	171.8 ± 6
EMT6						
Non-irradiated	61.3 ± 7.2^ab^	75 ± 5.7	22.0 ± 3.0^ab^	16.6 ± 2.3	94.3 ± 3.6	2396.0 ± 159.0
Irradiated	263.6 ± 45.1	93.1 ± 5.3	30.5 ± 4.0^bc^	49.9 ± 3.3	164.1 ± 12.5	4688.2 ± 99.9
% change	356.6 ± 7	19.1 ± 13.5	22.5 ± 8.5	203 ± 36.1	76.8 ± 2.8	102.7 ± 7.5

The 4T1 and EMT6 cells were exposed to 0 Gy (non-irradiated) or 100 Gy. At 24 h after irradiation, cells were harvested and stained with fluorochrome conjugated anti-CD80 (B7–1), anti-CD86 (B7–2), anti-H2-K^b^ (MHC I), anti-I-A^d^/I-E^d^ (MHC II), anti-CD54 (ICAM-1), and anti-CD95 (FasR). The samples were analyzed on a multiparameter flow cytometer (FACSCantoII). The differences in mean fluorescence intensities, ΔMFI, between unstained and stained non-irradiated cells, and unstained and stained irradiated cells were tabulated. The experiment was repeated twice and one representative experiment with triplicates is shown. The results are represented as ΔMFI ± standard deviation. Means with the same letter are not statistically significant from each other (*p* > 0.05, two-way analysis of variance followed by Tukey’s multiple comparison)

B7–1 and B7–2 are costimulatory molecules that bind to CD28 on T cells. This binding provides a second signal that is required for generation of an adaptive immune response. Prior to irradiation, though the level of B7–1 expressed by EMT6 cells was higher than 4T1 cells, the difference was not significant. (Table 1). However, upon irradiation, the ΔMFI value of EMT6 cells (263.6 ± 45.1) was significantly higher than 4T1 cells (14.8 ± 2.8). On the other hand, the ΔMFI value of B7–2 for EMT6 cells were significantly higher than 4T1 cells, before and after irradiation.

ICAM-1 is a ligand for LFA-1, which is expressed on a number of cell types, including T cells, APCs and some cancer cells. In general, the high levels of expression of ICAM-1 by cancer cells could promote an increased level of transcellular migration of leukocytes to the tumor site. Additionally, ICAM-1 expression also acts as a costimulatory signal for CTL activation [63]. Our analysis found no significant difference in the ΔMFI values of ICAM-1 between 4T1 and EMT6 before and after irradiation (Table 1).

FasR is a death receptor, which when bound to Fas-L on CTLs, can cause apoptosis of the cell expressing FasR. The ΔMFI value of FasR for EMT6 cells was significantly higher than that of 4T1 cells, both before and after irradiation. Specifically, ΔMFI of FasR for 4T1 cells were only 405.3 ± 20.4 and 1073.0 ± 71.6, compared to 2396.0 ± 159.0 and 4688.2 ± 99.9 for EMT6 cells, before and after irradiation, respectively (Table 1).

### Differences in cytokine release

**Fig. 3:**
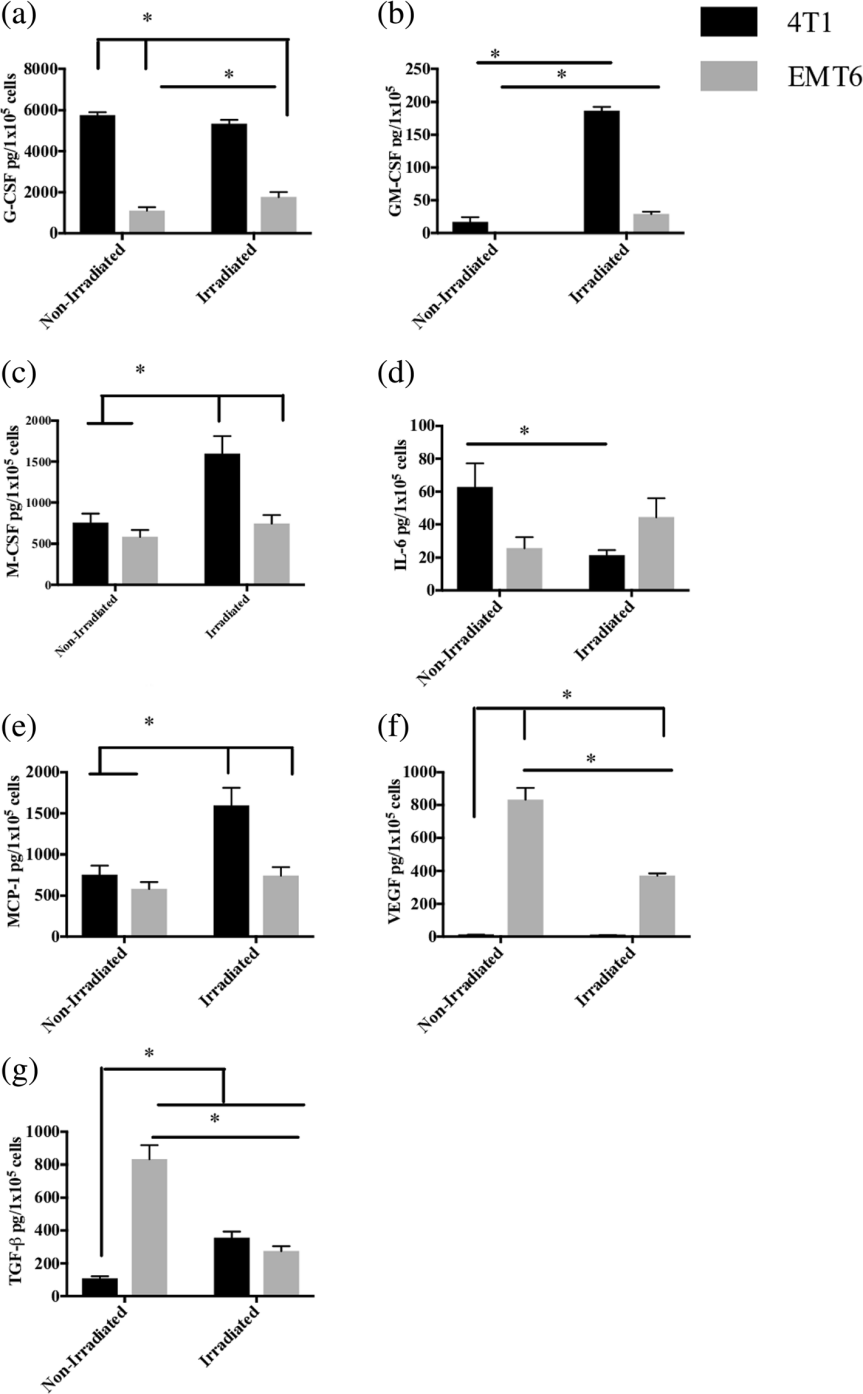
Cytokine release profile of 4T1 and EMT6 cells before and after irradiation. The 4T1 or EMT6 cells were exposed to 0 Gy (non-irradiated) or 100 Gy. The cells (5 × 10^5^ non-irradiated (4T1 and EMT6) and irradiated (4T1 Irr and EMT6 Irr) cells) were seeded on separate T25 flasks and cultured for 48 h. Cell culture supernatants were collected from each flask and centrifuged to remove debris. Levels of IL-6 (**a**), granulocyte-macrophage colony-stimulating factor (GM-CSF) (**b**), monocyte chemotactic protein-1 (MCP-1) (**c**), and granulocyte colony-stimulating factor (G-CSF) (**d**) in cell-free supernatants were quantified using cytometric bead array. Levels of macrophage colony-stimulating factor (M-CSF) (**e**), vascular endothelial growth factor (VEGF) (**f**), and tumor growth factor-beta (TGF-β) (**g**) were quantified by ELISA. The experiment was repeated thrice and the results represent the mean ± standard error from one representative experiment (*p < 0.05, two-way analysis of variance with Tukey’s multiple comparisons post-hoc analysis)

Another factor that could influence the immunogenicity of a tumor cell vaccine is its spontaneous release of cytokines and growth factors. To this end, IL-6, VEGF, TGF-β, MCP-1 and colony stimulating factors G-CSF, M-CSF and GM-CSF secreted by 4T1 and EMT6, before and after irradiation were compared (Fig. 3).

Colony stimulating factors, G-CSF, GM-CSF and M-CSF, at physiological levels, stimulate the proliferation, differentiation and survival of immune-supporting myeloid cells. However, at higher levels, these growth factors are associated with aberrant myeloid differentiation and accumulation of immunosuppressive MDSCs [64]. All three colony stimulating factors were produced at significantly higher levels by irradiated 4T1 cells compared to irradiated EMT6 cells (Fig. 3a, b, c). Most strikingly, the levels of G-CSF released by 4T1 cells before irradiation (5765 ± 80.9 pg/10^5^ cells) and after irradiation (5334 ± 114.2 pg/10^5^ cells) were exceptionally high even when compared to the levels released by EMT6 cells before (1100 ± 98.84 pg/10^5^ cells) and after irradiation (1760 ± 145.1 pg/10^5^ cells) (Fig. 3a).

IL-6 has been shown to exhibit both pro- and anti-tumor activities. Among its suppressive activities, IL-6 directly promotes cancer cell proliferation, survival and metastasis while indirectly supporting angiogenesis in the tumor microenvironment [65]. Here, IL-6 was released at modest levels with no major changes before irradiation (4T1: 62.6 ± 8.4 pg/10^5^ cells; EMT6: 25.6 ± 3.7 pg/10^5^ cells) or after irradiation (4T1: 21.3 ± 1.8 pg/10^5^ cells; EMT6: 44.3 ± 6.6 pg/10^5^ cells) (Fig. 3d).

Tumor-derived MCP-1 promotes infiltration of monocytes and macrophages. MCP-1 as well as VEGF are associated with promoting angiogenesis [66, 67]. Additionally, tumor secretion of VEGF blocks normal myeloid differentiation, resulting in MDSC accumulation [68, 69]. 4T1 cells produced higher levels of MCP-1 only after irradiation (4T1: 1596 ± 123.6 pg/10^5^ cells; EMT6: 744.7 ± 58.91 pg/10^5^ cells) (Fig. 3e). On the contrary, EMT6 cells produced higher levels of VEGF before (833 ± 41.19 pg/10^5^ cells) and after (371.3 ± 8.09 pg/10^5^ cells) irradiation, when compared to 4T1 cells before (10 ± 1.1 pg/10^5^ cells) and after (8.6 ± 0.6 pg/10^5^ cells) (Fig. 3f).

TGF-β, an immunosuppressive cytokine that plays a role in the induction of T_regs_, was produced at higher levels by EMT6 cells before irradiation (4T1: 108 ± 7.6 pg/10^5^ cells; EMT6: 832 ± 49 pg/10^5^ cells) (Fig. 3g). However, upon irradiation, the difference between cell lines was not statistically significant (4T1: 355 ± 22.1 pg/10^5^ cells; EMT6: 274 ± 17 pg/10^5^ cells) (Fig. 3g).

### Local and systemic effects of 4T1 mediated immunosuppression

**Fig. 4:**
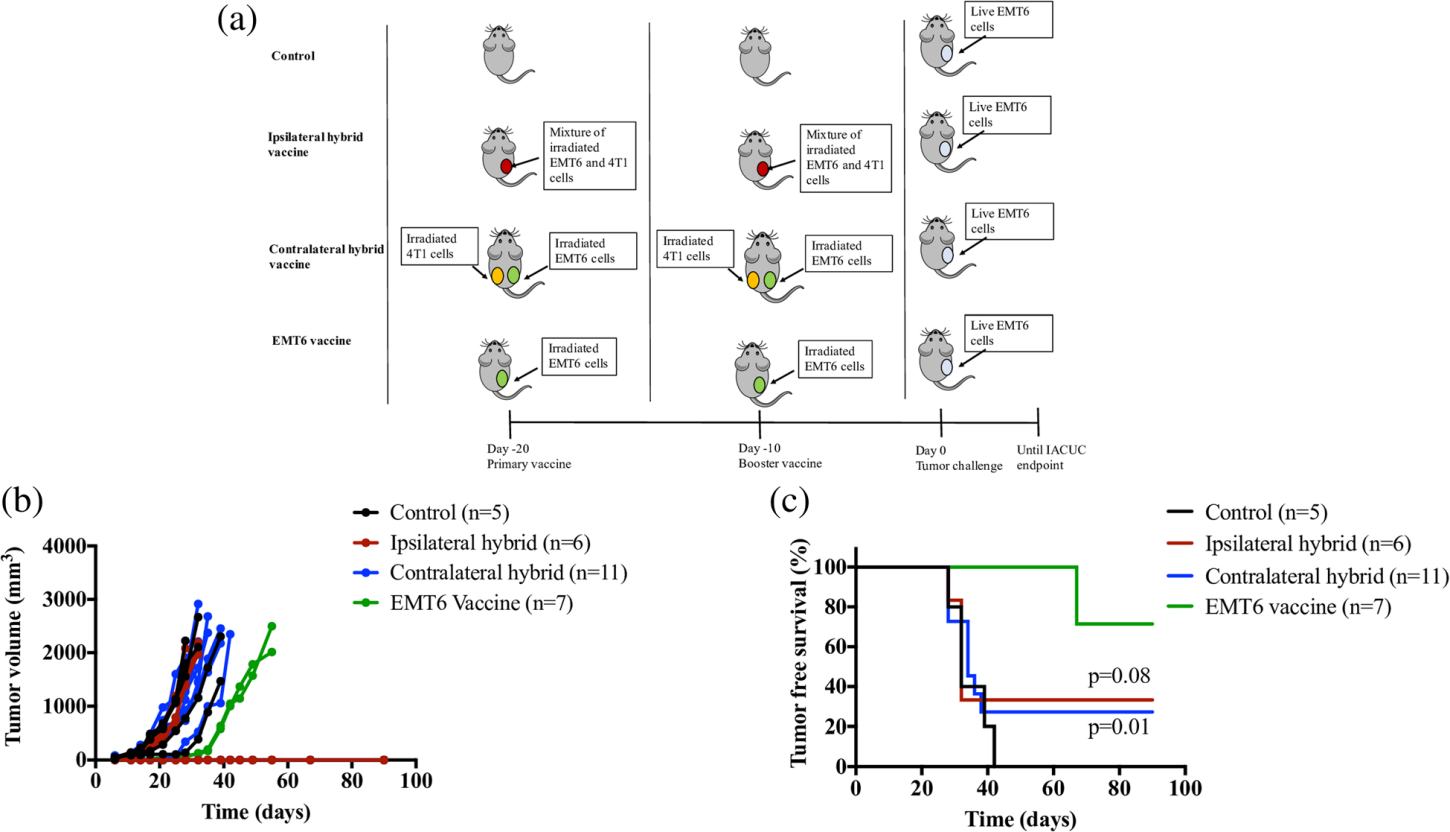
The 4T1 vaccine abrogates EMT6 immunity. **a** Female balb/cByJ mice were vaccinated with irradiated 5 × 10^5^ EMT6 cells (EMT6 vaccine) or a homogenous mixture of irradiated 5 × 10^5^ EMT6 and 1 × 10^6^ 4T1 cells (ipsilateral hybrid vaccine) or irradiated 1 × 10^6^ 4T1 and 5 × 10^5^ EMT6 on opposite flanks (contralateral hybrid vaccine) twice, 10 days apart. Ten days after the booster vaccine, all mice were challenged with 5 × 10^5^ live EMT6 cells on the same side as the irradiated EMT6 cells. Unvaccinated naïve mice challenged with 5 × 10^5^ live EMT6 cells served as controls. Tumor volumes (**b**) and tumor-free survival (**c**) were recorded. Differences in survival were compared determined by log-rank analysis. IACUC, Institutional Animal Care and Use Committee

Based on differences in cytokine release (Fig. 3), we explored if immunosuppressive cytokines released by 4T1 cells would abrogate the protective immunity established by the irradiated EMT6 vaccine. To explore the potential for localized immune suppression, mice were immunized with a heterogeneous mixture of irradiated 4T1 and EMT6 cells (ipsilateral hybrid vaccine). To explore possible systemic immune suppression mediated by 4T1 cells, mice were vaccinated with irradiated 4T1 cells and irradiated EMT6 cells on opposite flanks (contralateral hybrid vaccine). The efficacy of these vaccines was compared using mice immunized with irradiated EMT6 cells alone (EMT6 vaccine). The study design is shown in Fig. 4a.

When all groups of mice were challenged with live EMT6 cells, unlike the EMT6 vaccine group, both ipsilateral and contralateral hybrid vaccine group did not exhibit a delayed tumor incidence (Fig. 4b). Additionally, the presence of irradiated 4T1 cells in the hybrid vaccines diminished the protective immunity induced by irradiated EMT6 cells, as the percentage of tumor free survival in the ipsilateral and contralateral vaccine groups were only about 34% and 27%, respectively (Fig. 4c). The long term, tumor-free survival for mice receiving the EMT6 vaccine alone was significantly higher at 71%.

### The immunosuppressive role of G-CSF

Due to the exceptionally high levels of G-CSF produced by 4T1 cells with and without irradiation, we hypothesized that it played a key role in inhibiting the efficacy of ipsilateral and contralateral vaccines. To test this hypothesis, we functionally deleted the G-CSF gene via CRISPR/Cas9 genomic editing.

**Fig. 5:**
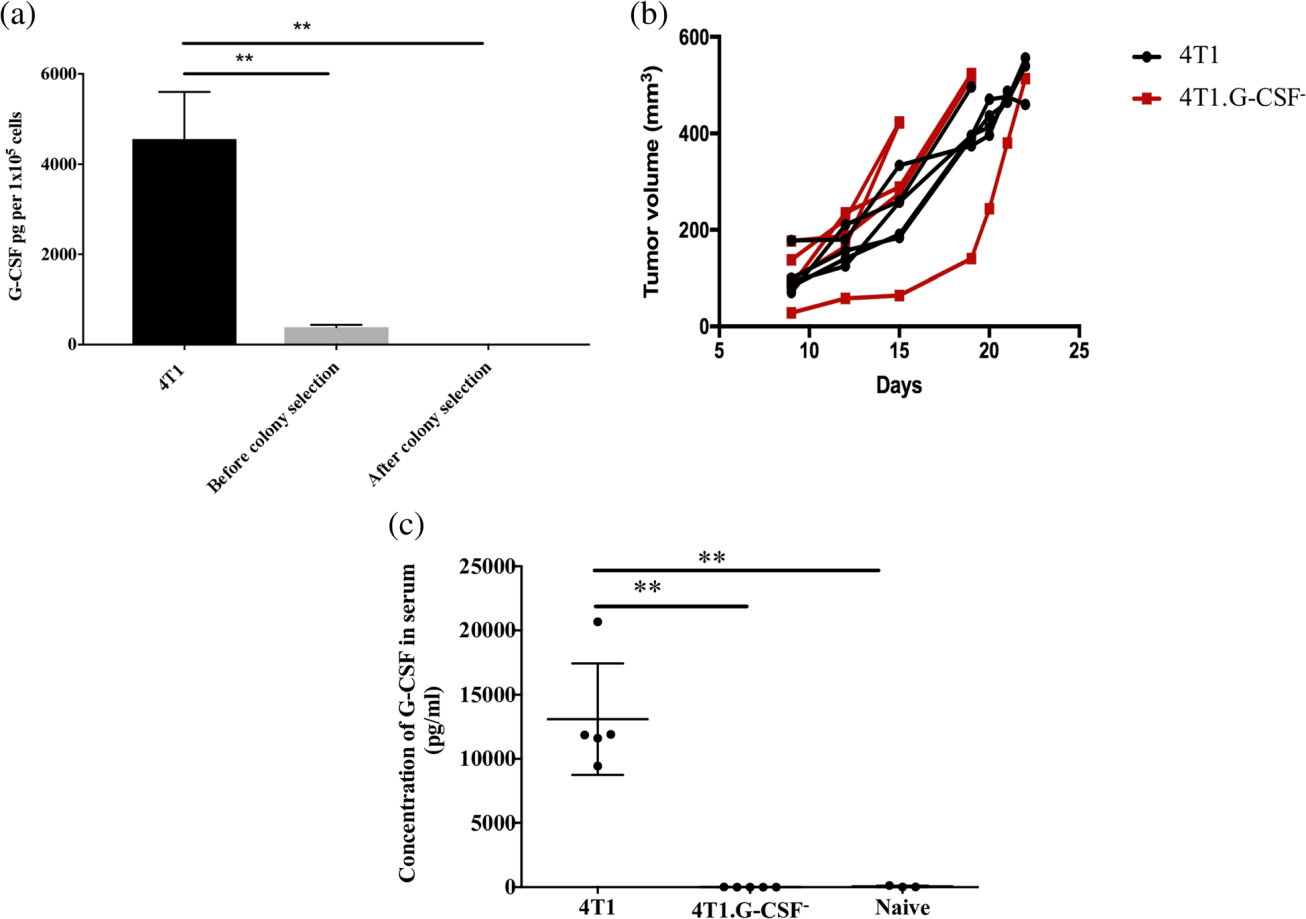
Effect of granulocyte colony-stimulating factor (G-CSF) functional deletion on G-CSF secretion and primary tumor growth. Cells (5 × 10^5^ 4T1 and 4T1.G-CSF^−^ cells) before and after colony selection were plated on a 6-well plate and the supernatant was collected after 24 h in culture. The experiment was repeated three times. **a** The concentration of G-CSF in the supernatant was detected by ELISA. Female balb/cByJ mice received subcutaneous injections of 5 × 10^5^ 4T1 (*n* = 5) or 4T1.GCSF^−^ cells (n = 5). **b** Tumor volumes were recorded and blood was collected when tumors reached 500–700 mm^3^. Serum from naïve mice (*n* = 3) served as controls. **c** The concentration of G-CSF in serum was determined by ELISA (**p < 0.01 via one-way analysis of variance with Tukey’s post-hoc analysis)

4T1 cells before G-CSF deletion released 4550 ± 604 pg G-CSF per 10^5^ cells, whereas after G-CSF deletion, cells only released 386 ± 31 pg G-CSF/10^5^ cells. Clonal selection led to the propagation of a 4T1 colony, called 4T1.G-CSF^−^, that released lower than detectable levels of G-CSF in vitro (Fig. 5a). The G-CSF deletion did not affect tumor establishment or growth in vivo (Fig. 5b). To further verify G-CSF ablation, G-CSF serum concentrations were assessed in mice bearing 4T1.G-CSF^−^ tumors when tumor volumes reached 500–700 mm^3^. 4T1.G-CSF^−^ tumor bearing mice contained only 10 ± 2.9 pg/ml of G-CSF in their sera, which was comparable to G-CSF in the sera of naïve mice (59 ± 34 pg/ml). On the other hand, mice with similarly sized, unmodified 4T1 tumors contained 13,096 ± 1947 pg/ml G-CSF in their sera (Fig. 5c).

**Fig. 6:**
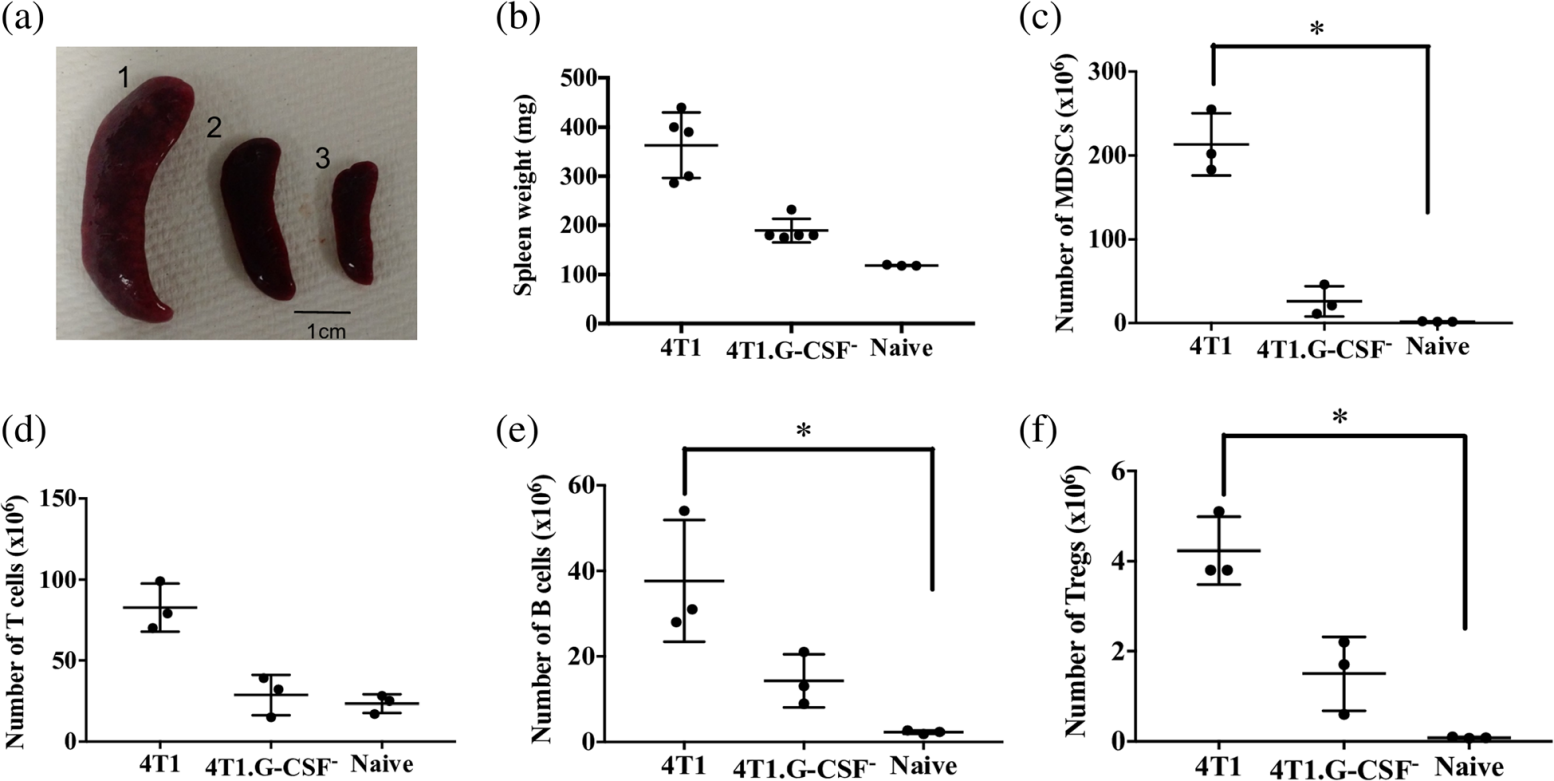
Comparison of spleen size, spleen weight and immune cell subsets in spleen of 4T1 and 4T1.G-CSF^−^ tumor-bearing mice and naïve mice. Female balb/cByJ mice received subcutaneous injections of 1 × 10^6^ 4T1 or 4T1.G-CSF^−^ cells. Spleens were harvested when tumor volumes reached 500–700 mm^3^. **a** Spleens from representative mice bearing (1) 4T1, (2) 4T1.G-CSF^−^ tumors and from (3) a naïve mouse. **b** Spleen weight at the time of harvest. The spleens were processed and flow cytometric analysis was performed to determine the percentage of myeloid derived suppressor cells (MDSCs) (CD11b^+^Ly6G^+^Ly6C^+^), B cells (CD19^+^), T cells (CD3^**+**^) and regulatory T cells (T_regs_) (CD4^+^CD25^+^FoxP3^+^). Absolute numbers of MDSCs (**c**), T cells **(d**), B cells (**e**), and T_regs_ (**f**) were quantified and results are presented as mean ± standard error (*p < 0.05, Kruskal-Wallis test with Dunn’s post-hoc test)

Additionally, spleens and DLNs from 4T1 and 4T1.G-CSF^−^ tumor bearing mice were harvested to determine the frequency of T cells, B cells, MDSCs and T_regs_ in each lymphoid tissue. MDSCs were of particular interest as immature myeloid cells have often been associated with high levels of colony stimulating factors [70, 71]. Prior to immunophenotyping we noted remarkable differences in the sizes and weights of spleens removed from mice bearing 4T1 tumors, 4T1.GCSF^−^ tumors or no tumors (Fig. 6a, b). G-CSF appeared to be driving the extreme splenomegaly observed in 4T1-bearing mice. In addition, spleens from 4T1 tumor bearing mice contained significantly higher levels of MDSCs (213 ± 21 × 10^6^ MDSCs) when compared to naive (1.7 ± 0.3 × 10^6^ MDSCs) spleens. On the other hand, there was no significant difference between the levels of MDSCs in naïve and 4T1.G-CSF^−^ tumor bearing mice (26 ± 10 × 10^6^ MDSCs)(Fig. 6c). The same trends held when analyzing the percentages of MDSCs in the spleens of mice from the three groups (see Additional file 1).

Though there was no significant difference in the number of T cells in the spleens of mice among the different groups, there was a significant difference in the number of B cells and T_regs_ between 4T1 tumor bearing and naïve mice (4T1: 37 ± 8 × 10^6^ B cells and 4.2 ± 0.5 × 10^6^ T_regs_; naïve: 2.3 ± 0.4 × 10^6^ B cells and 0.08 ± 0.01 × 10^6^ T_regs_) (Fig. 6d, e, f). Immune subset populations from 4T1.G-CSF^−^ tumor bearing mice were comparable with those found in naïve mice. Percentages of splenic T cells, B cells and T_regs_ were not statistically different among the cohorts (see Additional file 1).

**Fig. 7:**
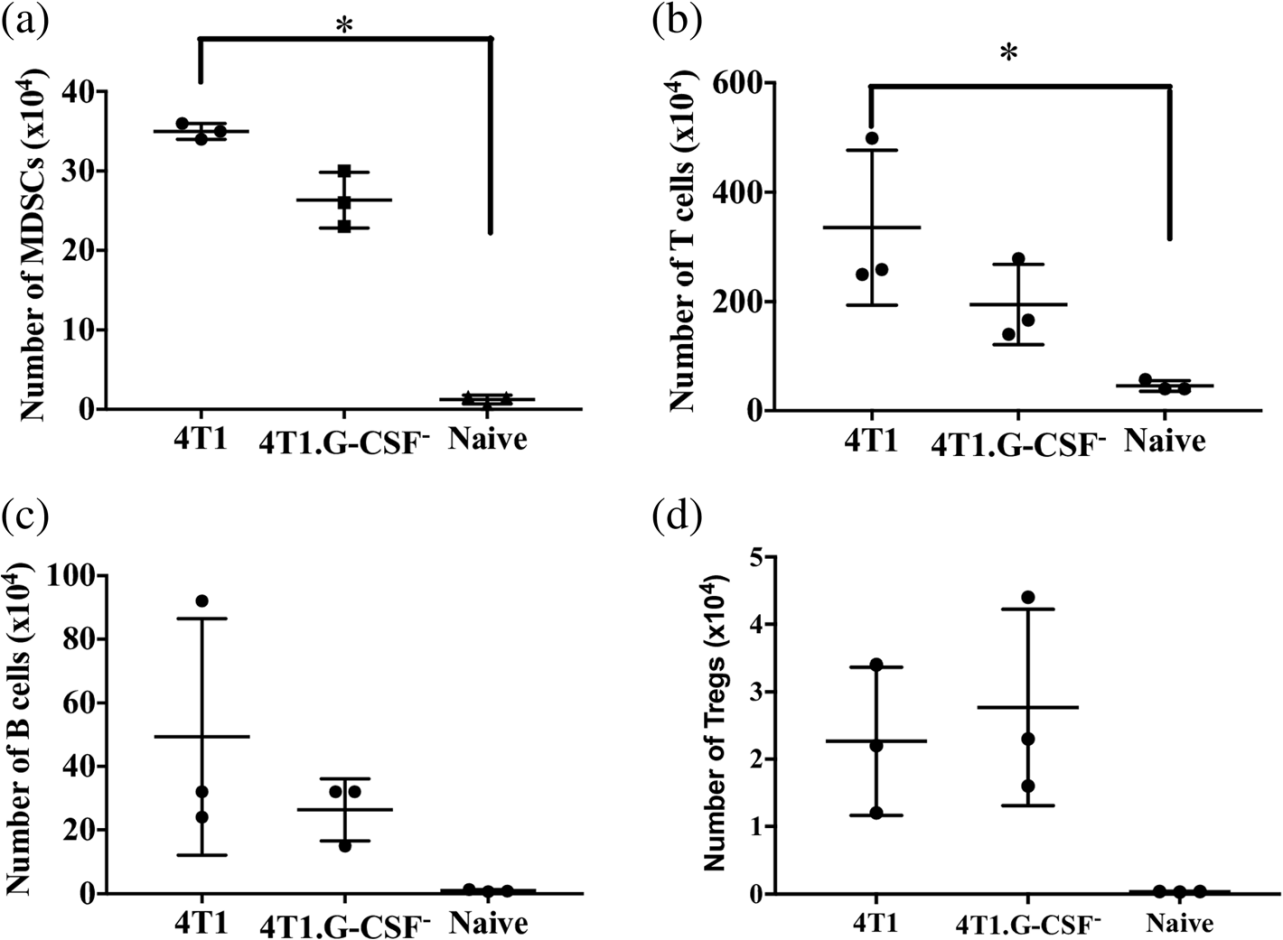
Comparison of immune cell subsets in draining lymph nodes (DLNs) from mice bearing 4T1 and 4T1.G-CSF^−^ and naïve mice. Female balb/cByJ mice (*n* = 3) received subcutaneous injections of 1 × 10^6^ 4T1 or 4T1.G-CSF^−^ cells. Once tumors reached 500–700 mm^3^, DLNs were harvested and single cell suspensions obtained. Leukocytes isolated from naïve mice served as control (*n* = 3). Flow cytometric analysis was performed to determine the percentage of myeloid derived suppressor cells (MDSCs) (CD11b^+^Ly6G^+^Ly6C^+^), B cells (CD19^+^), T cells (CD3^**+**^) and regulatory T cells (T_regs_) (CD4^+^CD25^+^FoxP3^+^). Absolute numbers of MDSCs (**a**), T cells **(b**), B cells (**c**), and T_regs_ (**d**) were quantified and results are represented as mean ± standard error (*p < 0.05, Kruskal-Wallis test with Dunn’s post-hoc test)

In DLNs, numbers of MDSCs in 4T1 tumor bearing mice (35 ± 0.5 × 10^4^ MDSCs) was significantly higher than the levels in naïve mice (1.2 ± 0.3 × 10^4^ MDSCs) but not 4T1.G-CSF^−^ tumor bearing mice (Fig. 7a). Similarly, DLNs contained significant differences in the numbers of T cells between 4T1 bearing and naïve mice, whereas the T cell levels were similar between 4T1.G-CSF^−^ and naïve mice (4T1: 335 ± 81 × 10^4^ T cells; 4T1.G-CSF^−^: 194 ± 42 × 10^4^ T cells; naïve: 45 ± 5 × 10^4^ T cells) (Fig. 7b). No significant differences in the numbers of B cells and T_regs_ between the three groups was observed (Fig. 7c, d). In terms of percentages, both 4T1 and 4T1.G-CSF^−^ tumor bearing mice had higher percentages of MDSCs in their DLNs than naïve mice, while there were no differences in T cell or B cell percentages (see Additional file 2).

### G-CSF secretion versus MDSC accumulation in different breast cancers

**Fig. 8:**
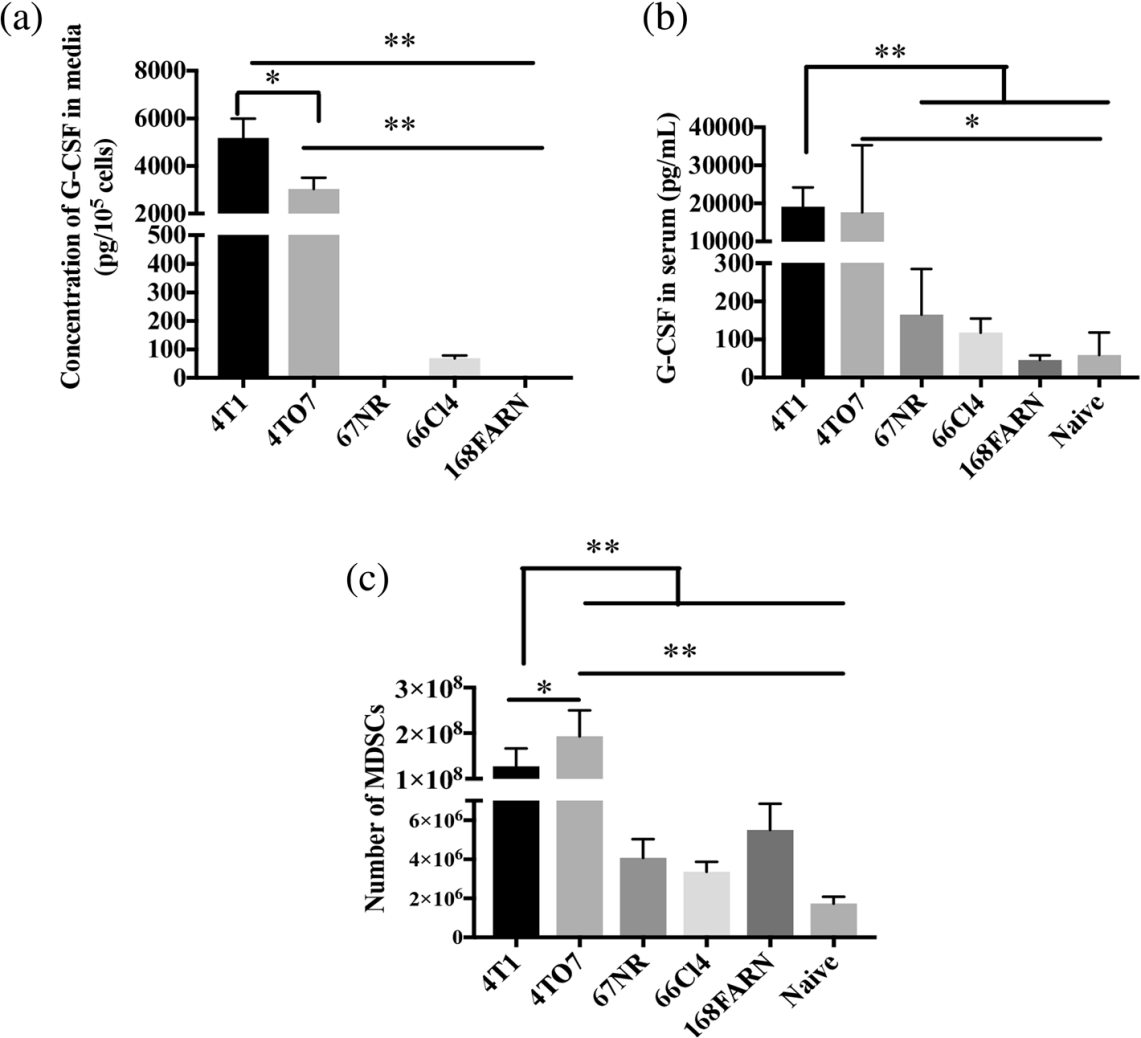
Concentration of granulocyte colony-stimulating factor (G-CSF) in culture (in vitro) and in serum (in vivo) in mice bearing different breast cancer cell lines compared to the number of myeloid derived suppressor cells (MDSCs) in spleen. **a** The 4T1, 4T07, 67NR, 66Cl4 and 168FARN were seeded on separate T25 flasks and cultured for 48 h. G-CSF concentrations from culture supernatants were determined by ELISA**. b** Balb/cByJ mice were subcutaneously injected with 1 × 10^6^ 4T1 cells (*n* = 5), 5 × 10^6^ 4T07 cells (*n* = 3), 1 × 10^6^ 168FARN (*n* = 3), 1 × 10^6^ 67NR (*n* = 5) and 3 × 10^6^ 66Cl4 (*n* = 5). When tumor volumes reached 500 mm^3^, blood and spleens were harvested. Serum G-CSF concentrations were determined by ELISA. **c** Absolute numbers of MDSCs (CD11b^+^Ly6G^+^Ly6C^+^) among splenocytes from the same tumor-bearing mice were determined by flow cytometry. Results are representative of two independent experiments. Data are presented as mean ± standard error (*p < 0.05, **p <30.01 via one-way analysis of variance with Tukey’s post-hoc analysis)

To further establish the correlation between tumor secreted G-CSF levels and MDSC accumulation, we used four different 4T1sister cell lines, namely 4 T07, 67NR, 66Cl4 and 168 FARN, that share a common origin, a single, spontaneously arising breast tumor, but differ in their metastatic ability [72]. 4T1 metastasizes to lung, liver, brain and bone; 66Cl4 metastasizes to lungs and liver; 168 FARN metastasizes to regional lymph nodes; whereas 67NR and 4T07 do not form visible metastases although 4T07 cells can be found in blood and lungs [73]. In vitro studies revealed that 4T1 and 4 T07 cells secreted high levels of G-CSF (4T1: 5182 ± 814 pg/10^5^ cells, 4T07: 3032 ± 476 pg/10^5^ cells), while 66Cl4 secreted only about 68.5 ± 10.6 pg/10^5^ cells. On the other hand, G-CSF secretion by 67NR and 168FARN cells was undetectable (Fig. 8a). Similarly, we found that serum G-CSF levels in mice bearing 4T1 and 4T07 tumors were much higher (4T1: 19,100 ± 2274 pg/ml, 4T07: 17,600 ± 10,220 pg/ml) than the other tumor models (Fig. 8b). mice bearing 67NR, 66Cl4, and 168FARN had only 165 ± 53 pg/ml, 117 ± 16 pg/ml and 46 ± 6 pg/ml, of serum G-CSF respectively. These levels were not significantly different from serum G-CSF in naïve mice (59 ± 34 pg/ml) (Fig. 8b). To determine the relationship between serum G-CSF levels and accumulation of MDSCs, cohorts of mice were implanted with each of the tumor cell lines and spleens harvested for enumeration of MDSCs. Spleens from 4T1 and 4T07 tumor-bearing mice contained 1.27 ± 0.1 × 10^8^ and 1.9 ± 0.3 × 10^8^ MDSCs, respectively. This was significantly higher MDSC accumulation compared to the remaining breast tumor models (67NR: 4 ± 0.4 × 10^6^; 66Cl4: 3 ± 0.2 × 10^6^; 168FARN: 5 ± 0.7 × 10^6^ MDSCs) (Fig. 8c).

### Effect of G-CSF on protective immunity

**Fig. 9:**
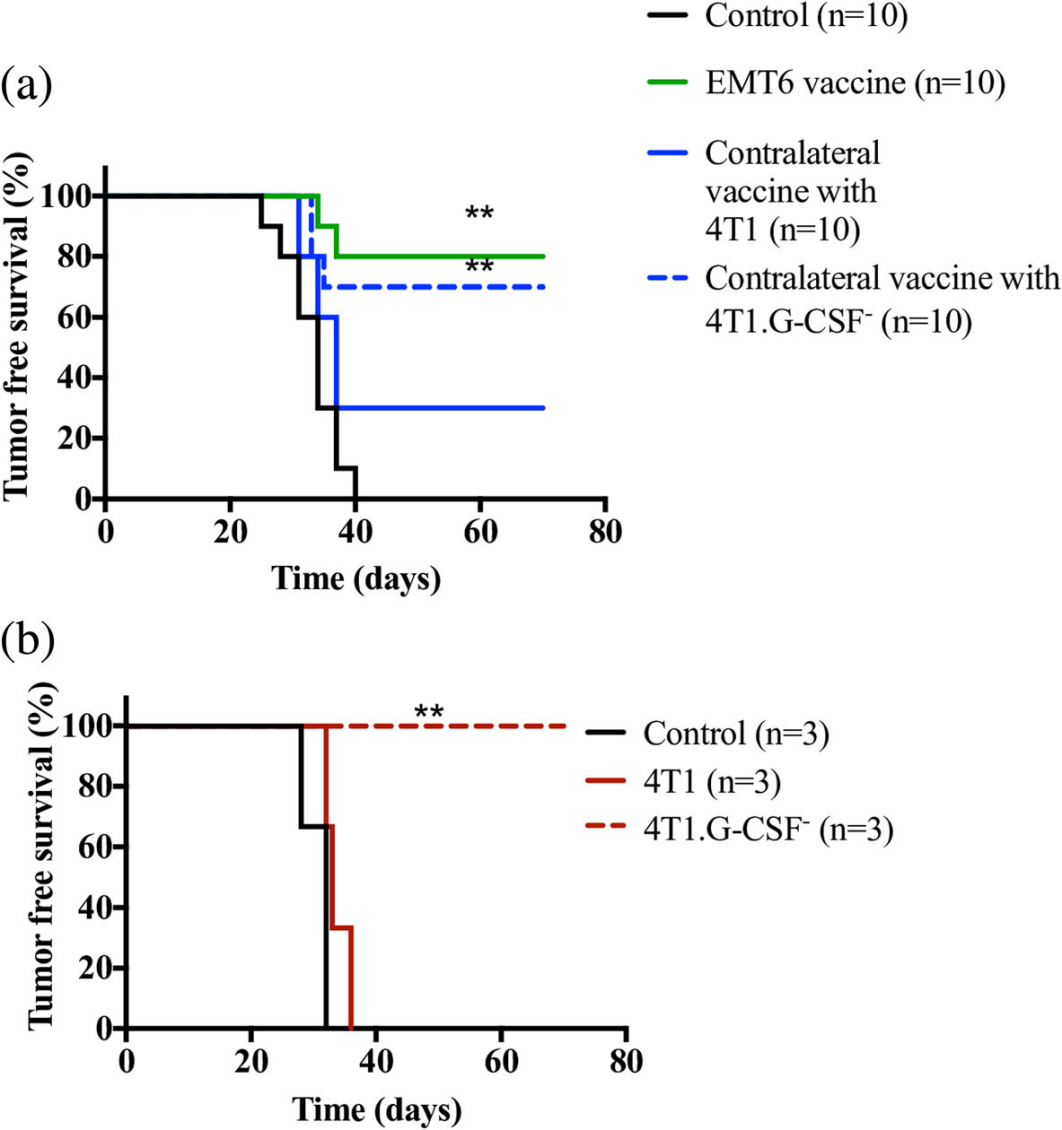
Prophylactic vaccination with 4T1.G-CSF^−^ cells. **a** Balb/cByJ mice received 5 × 10^5^ irradiated EMT6 cells or 5 × 10^5^ irradiated EMT6 cells and 1 × 10^6^ irradiated 4T1 cells on opposite sides (contralateral 4T1 vaccine) or 5 × 10^5^ irradiated EMT6 cells and 1 × 10^6^ irradiated 4T1.G-CSF^−^ cells on opposite sides (contralateral 4T1.G-CSF^−^ vaccine) twice, 10 days apart. Ten days after the booster vaccination, all mice were challenged with 5 × 10^5^ live EMT6 cells. Additionally, naive mice that received only 5 × 10^5^ live EMT6 cells served as controls. Tumor-free survival was recorded. **b** Balb/cByJ mice were vaccinated with 1 × 10^6^ 4T1 cells or 1 × 10^6^ 4T1.G-CSF^−^ cells, twice 10 days apart. Ten days after the booster vaccination, mice were challenged with live 5 × 10^5^ 4T1 cells or 5 × 10^5^ 4T1.G-CSF^−^ cells, respectively. Naive mice that received only 5 × 10^5^ live 4T1 cells, served as controls. Tumor-free survival was recorded (**p < 0.01, log-rank analysis)

From the aforementioned studies, it appeared that MDSCs arising from high levels of G-CSF released by 4T1 vaccine were directly responsible for the impaired response to the EMT6 vaccine. To test this hypothesis, we repeated the contralateral hybrid vaccine study (Fig. 4) with irradiated 4T1.GCSF^−^ plus EMT6 cells followed by a live EMT6 challenge. We found that only 30% of mice receiving a hybrid vaccine containing 4T1.G-CSF^−^ cells developed and succumbed to tumors (Fig. 9a). This was significantly different from a hybrid vaccine containing parental 4T1 cells, where 70% of the mice developed tumors (Fig. 9a). Additionally, we recorded survival in mice that were vaccinated and challenged with 4T1 or 4T1.G-CSF^−^ cells alone, i.e. without including EMT6 cells. We found that in the 4T1 group, all mice developed tumors similar to unvaccinated controls (Fig. 9b). On the other hand, none of the mice in the 4T1.G-CSF^−^ vaccine group developed tumors (Fig. 9b). Thus, deleting G-CSF in 4T1 cells appears to render them more immunogenic than EMT6 cells (Figs. 2 and 4).

## Discussion

A major barrier to the widespread development of ATCVs as an adjuvant to breast tumor resection is the sporadic, often poor, immunogenicity of resected breast cancer cells. Thus, in this study, we set out to determine the key factors influencing the immunogenicity of breast cancer ATCVs by comparing two murine breast cancer cells, 4T1 and EMT6. This study was not meant to provide a comprehensive account of immunogenic and immunosuppressive elements in breast cancer, but rather a springboard for further exploration of key factors in other tumor models and clinical samples.

Though the use of irradiated tumor cells to develop autologous tumor cell vaccines is reported extensively in the literature, we first wanted to demonstrate that we could effectively inactivate 4T1 and EMT6 cells using this approach. We tested for the effect of different doses of irradiation (20, 40, 60, 80, and 100 Gy) on tumor cell proliferation using a proliferation assay as described in “Methods”. We confirmed that at all five doses, both 4T1 and EMT6 cells failed to proliferate over the observed period of 4 days (Fig. 1). Thus, for experiments throughout this study, we chose 100 Gy, the highest dose tested, as a standard to develop our tumor cell vaccines.

The 4T1 cell line is often referred to as poorly immunogenic, while EMT6 is considered as a highly immunogenic cell line. Since the immunogenicity of these two cell lines has never been directly compared in any study, we first confirmed the differences in their immunogenicity in the ATCV setting. We vaccinated mice with irradiated 4T1 or EMT6 cells and subsequently challenged with live 4T1 or EMT6 cells. We found that mice immunized with EMT6 cells developed partial-to-complete protective immunity against live EMT6 challenge. On the other hand, 4T1 vaccine failed to provide any measurable protective immunity (Fig. 2). These data were consistent with previous publications [54, 74].

To explore potential causes of immunogenic differences, we first looked at the expression of the immunologically relevant surface molecules MHC I, MHC II, B7–1, B7–2, ICAM-1, and FasR. We found that irradiated EMT6 cells express significantly higher levels of MHC I, B7–1, B7–2, and FasR, which could be responsible for the enhanced immune response to EMT6 vaccine (Table 1). We next analyzed cytokines released by each cell line and found that irradiated 4T1 cells released very high levels of GM-CSF, M-CSF, and in particular G-CSF (Fig. 3). Each of these cytokines can be immune-activating or immune-suppressive depending on their concentrations and context [75–78].

The hybrid vaccine studies (Fig. 4) were designed to help tease out the relative contributions of cell surface markers versus cytokines on ATCV responses. The 4T1 vaccine was unlikely to improve the activity of the EMT6 vaccine as the former was found to be non-immunogenic. However, if the 4T1 vaccine had no effect on the EMT6 vaccine, or if the 4T1 vaccine reduced the efficacy of the EMT6 vaccine only locally, i.e. in the ipsilateral setting, then decreased costimulation (signal 2) on the part of the 4T1 vaccine could have induced a localized tolerogenic effect. One also could have argued that local immunosuppressive cytokines may have inhibited the EMT6 vaccine as well. Conversely, if the 4T1 vaccine inhibited the EMT6 vaccine both locally and systemically, as was observed, then a soluble factor secreted at high levels by 4T1 cells must be responsible for the inhibited EMT6 vaccine response.

Of the different cytokines released by 4T1 cells, G-CSF was produced at exceptional levels (Fig. 3). At such high levels, G-CSF and other colony stimulating factors have been associated with MDSC expansion and immune impairment [71, 79–82]. Not surprisingly, we found that MDSC levels more or less correlated with G-CSF concentrations produced by five sister breast cancer cell lines: 4T1, 4T07, 67NR, 66Cl4 and 168FARN (Fig. 8). Although they did not measure cytokine production, Talmadge et al., also found significant differences in MDSC frequency among different breast tumor models, with 4T1 inducing the highest levels [83]. It should also be noted that, although other publications have suggested a link between G-CSF and/or MDSCs and tumor metastasis [64, 80, 84, 85], mice bearing 4T07 tumors, which do not induce visible metastatic lesions [73], produced high levels of G-CSF and more splenic MDSCs than mice bearing any other tumor including highly metastatic 4T1 or 66CL4 tumors. These data suggest that other factors, such as IL-6 [86], may be involved in breast cancer metastasis. At the very least, the relationship between G-CSF/MDSCs and metastasis may be model-dependent.

The correlation between G-CSF and MDSC accumulation was further solidified by functional deletion of G-CSF. Mice with 4T1 tumors, when compared to mice with 4T1.G-CSF^−^ tumors, had increased amounts of MDSCs in both spleens and DLNs (Figs. 6, 7). It should be noted that relatively high levels of CD11b^+^Ly6G^+^Ly6C^+^ cells were found in the DLNs of mice bearing 4T1.G-CSF tumors (Fig. 7). We did not evaluate the immunosuppressive activity of these cells, but it is unlikely that they were highly suppressive, if at all, given that mice vaccinated with 4T1.G-CSF^−^ cells were protected from a 4T1.G-CSF^−^ tumor challenge. This is a reminder that MDSCs are a diverse family of cells and that not all CD11b^+^Ly6G^+^Ly6C^+^ can be classified as MDSC [87]. While there were other differences in immune subset populations between 4T1 and 4T1.G-CSF^−^ tumor-bearing mice (Figs. 6, 7), similar to differences in costimulatory molecules, these were overshadowed by the enormous differences in the MDSC populations. To verify that tumor-derived G-CSF was responsible for abrogating vaccine efficacy, we repeated the hybrid vaccine study utilizing 4T1.G-CSF^−^ cells in the contralateral vaccine group. We found that the hybrid vaccine containing 4T1.G-CSF^−^ cells was far less immunosuppressive than the hybrid vaccine containing parental 4T1 cells (Fig. 9). Overall, the findings from this study establish a causal link between tumor-derived G-CSF and a loss of responsiveness to breast ATCVs.

As mentioned previously, the finding that 4T1-derived G-CSF leads to MDSC accumulation and immune suppression is not novel. However, that this immune impairment can be eliminated by knocking out a single, non-essential protein, G-CSF, despite an otherwise aggressive phenotype was somewhat surprising. In fact, it should be noted that after G-CSF deletion, the 4T1.G-CSF^−^ vaccine was more immunogenic and more protective than the EMT6 vaccine (Fig. 9b versus Figs. 2, 4). These data imply that tumor cell surface phenotype is not as important as tumor-derived secreted factors when establishing breast ATCV immunogenicity.

Although this study has provided useful insight into the effect of tumor-derived factors on ATCV efficacy, we acknowledge that it has a few limitations and opportunities for additional exploration. First, through the entirety of the study, tumor cell vaccines were only used in a prophylactic setting. To truly recapitulate the effect of the tumor-derived factors, future studies ought to focus on vaccinating the mice post tumor resection. Second, this study only used the 4T1 cell line to establish the causal link between G-CSF secretion and ATCV efficacy. Future studies that involve either knocking in or knocking out G-CSF in the other cancer cell lines that intrinsically secrete low or high levels of G-CSF will further strengthen the findings of this study. Third, there is no question that G-CSF-induced MDSCs are responsible for vaccine impairment in our models. However, G-CSF could also be causing immune suppression through additional pathways. In a recent clinical study, G-CSF was highly expressed in tumors in patients with breast cancer with more aggressive disease and was correlated with poorer overall survival [88]. As this study illustrates, tumor-associated macrophages (TAMs) are another key immunosuppressive subset that is strongly influenced by G-CSF. Assessing differences in TAM number and function between 4T1 and 4T1.G-CSF^−^ tumors is the subject of ongoing research. Likewise, while G-CSF is clearly an important target in breast cancer, it is important to note that our findings do not eliminate the possibility of other mechanisms that could be involved in MDSC expansion. For instance, knocking out other colony stimulating factors such as GM-CSF may have a similar effect on vaccine efficacy. Last, given that G-CSF and GM-CSF are routinely administered to prevent neutropenia in patients with breast cancer undergoing chemotherapy, a closer look at the immunosuppressive impacts of these cytokines, particularly in a setting of minimal residual disease, is warranted.

## Conclusion

ATCVs represent a safe and potentially effective weapon to prevent breast cancer recurrence in a patient-specific manner. A better understanding of factors that influence the effectiveness of breast cancer ATCVs will facilitate continued development and eventual clinical application. Here, our study began with an initial comparison of surface marker expression between a non-immunogenic and an immunogenic breast cancer cell line. While some differences were noted, the most obvious being the increase in B7–1 on the surfaces of EMT6 cells, these differences were far less striking when compared to differences in cytokine expression levels. The exceptionally high levels of G-CSF released by 4T1 cells were found to be responsible for MDSC accumulation, splenomegaly, and the associated abrogation of EMT6 vaccine responses. After knocking out G-CSF expression, 4T1 cells became as immunogenic as EMT6 cells in a prophylactic vaccination-tumor challenge experiment. These results imply that similar inhibition of immunosuppressive signals may help enhance, if not standardize, the immunogenicity of breast ATCVs.

## Additional files

Additional file 1: **Figure S1.** Comparison of percentage of immune cell subsets in spleen of 4T1 and 4T1.G-CSF^–^ tumor-bearing mice and naïve mice. (PDF 60 kb)

Additional file 2: **Figure S2.** Comparison of percentage of immune cell subsets in DLNs of 4T1 and 4T1.G-CSF^–^ tumor-bearing mice and naïve mice (PDF 60 kb)

## Abbreviations

ANOVA: Analysis of variance
APC: Antigen presenting cell
ATCV: Autologous tumor cell vaccine
CBA: Cytokine bead array
CRISPR: Clustered regularly interspaced short palindromic repeats
DLN: Draining lymph node
ELISA: Enzyme-linked immunosorbent assay
FasR: Fas receptor
G-CSF: Granulocyte colony-stimulating factor
GM-CSF: Granulocyte-macrophage colony-stimulating factor
ICAM-1: Intracellular adhesion molecule-1
IDO: Indoleamine-pyrrole 2,3-dioxygenase
IL-10: Interleukin-10
IL-6: Interleukin-6
LFA-1: Lymphocyte function-associated antigen-1
MCP-1: Monocyte chemotactic protein-1
M-CSF: Macrophage colony-stimulating factor
MDSC: Myeloid-derived suppressor cell
MFI: Median fluorescence intensity
MHC: Major histocompatibility complex
PD-1: Programmed cell death protein 1
PD-L1: Programmed death-ligand 1
TAM: Tumor associated macrophage
TCR: T cell receptor
TGF-b: Tumor growth factor-beta
TNBC: Triple-negative breast cancer
T_reg_: Regulatory T cell
VEGF: Vascular endothelial growth factor
