# Supplementary material for: Tumor-derived granulocyte colony-stimulating factor diminishes efficacy of breast tumor cell vaccines
Source: Breast Cancer Res. 2018 Oct 22;20:126. doi: 10.1186/s13058-018-1054-3 (PMC6198508; doi:10.1186/s13058-018-1054-3)
Supplement: Supplementary file 1 — Figure S1. Comparison of percentage of immune cell subsets in spleen of 4T1 and 4T1.G-CSF– tumor-bearing mice and naïve mice. (PDF 60 kb) [file 13058_2018_1054_MOESM1_ESM.pdf]

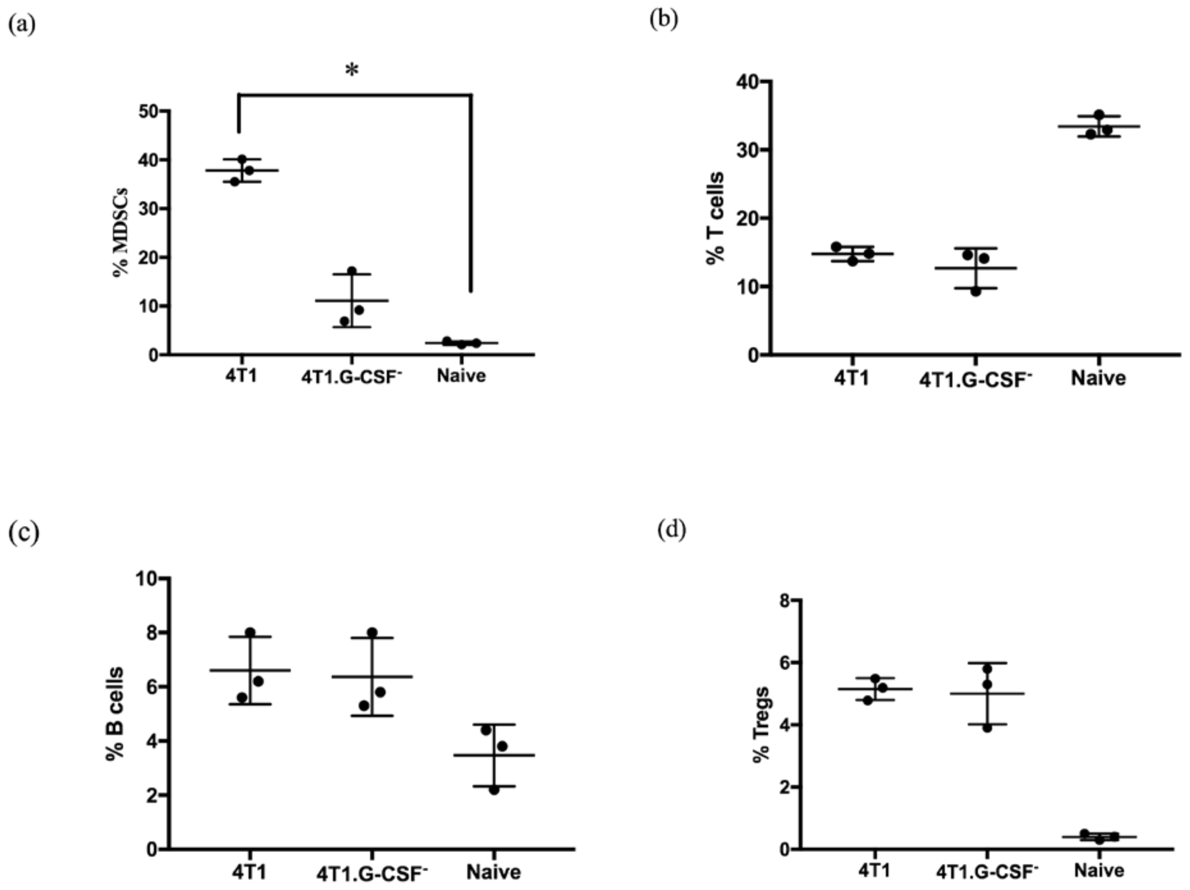

Figure S1. Comparison of percentage of immune cell subsets in spleen of 4T1 and 4T1.G-CSF^{-} tumor bearing mice with naïve mice. Female balb/cByJ mice received s.c. injections of $1x10^{6}$ 4T1 or 4T1.G-CSF^{-} cells. Spleens were harvested when tumor volumes reached 500-700mm^{3}. The spleens were processed and flow cytometric analysis was performed to determine the percentage of (a) MDSCs (CD11b^{+}Ly6G^{+}Ly6C^{+}), (b) B cells (CD19^{+}), (c) T cells (CD3^{+}) and (d) regulatory T cells (CD4^{+}CD25^{+}FoxP3^{+}). The data are presented as mean ± standard error (*$p<0.05$, via Kruskal-Wallis test with Dunn's post-hoc test).
