# Supplementary material for: Tumor-derived granulocyte colony-stimulating factor diminishes efficacy of breast tumor cell vaccines
Source: Breast Cancer Res. 2018 Oct 22;20:126. doi: 10.1186/s13058-018-1054-3 (PMC6198508; doi:10.1186/s13058-018-1054-3)
Supplement: Supplementary file 2 — Figure S2. Comparison of percentage of immune cell subsets in DLNs of 4T1 and 4T1.G-CSF– tumor-bearing mice and naïve mice (PDF 60 kb) [file 13058_2018_1054_MOESM2_ESM.pdf]

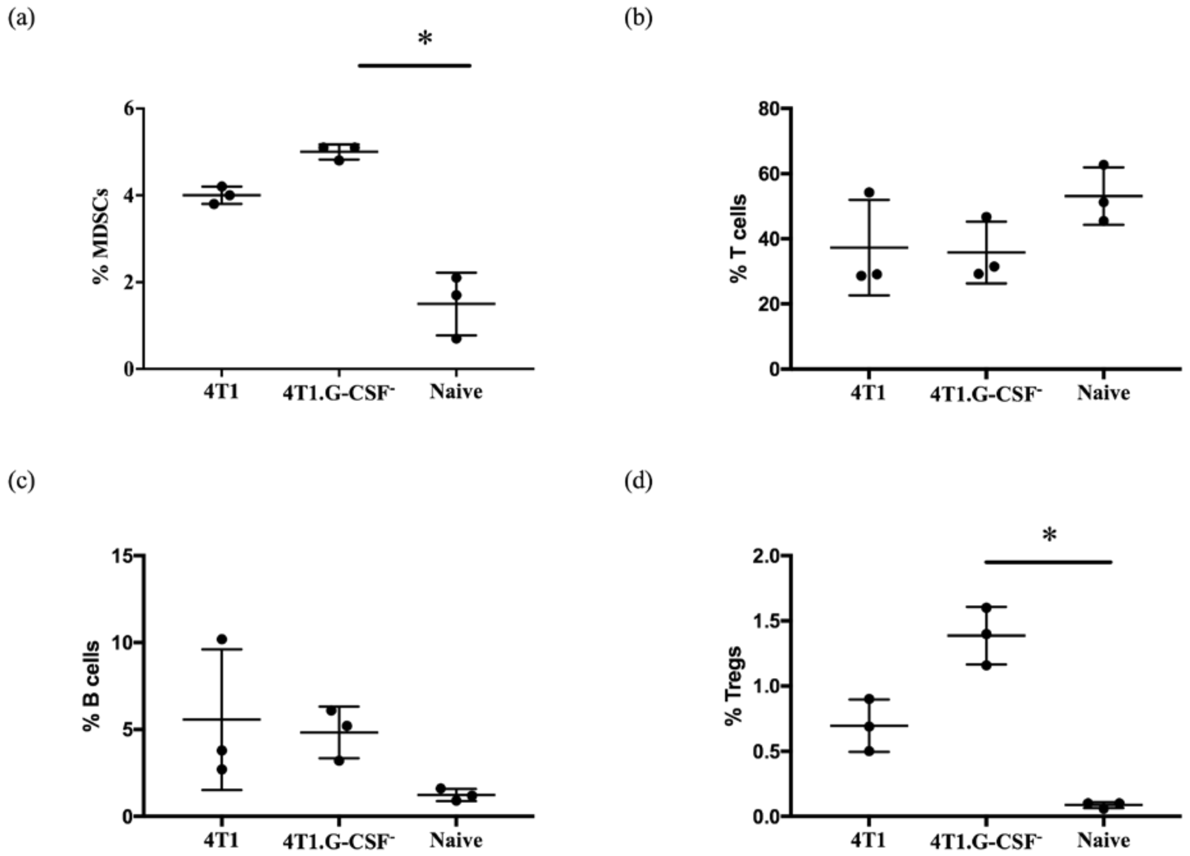

Figure S2. Comparison of percentage of immune cell subsets in DLNs of 4T1 and 4T1.G-CSF$^-$ tumor bearing mice with naïve mice. Female balb/cByJ mice received s.c. injections of 1x10^6 4T1 or 4T1.G-CSF$^-$ cells. DLNs were harvested when tumor volumes reached 500-700mm^3 and single suspensions were obtained. Flow cytometric analysis was performed to determine the percentage of (a) MDSCs (CD11b$^+$Ly6G$^+$Ly6C$^+$), (b) B cells (CD19$^+$), (c) T cells (CD3$^+$) and (d) regulatory T cells (CD4$^+$CD25$^+$FoxP3$^+$). The data are presented as mean ± standard error (*p<0.05, via Kruskal-Wallis test with Dunn's post-hoc test).
